# New Insights into Measurement Variability in Glaucomatous Visual Fields from Computer Modelling

**DOI:** 10.1371/journal.pone.0083595

**Published:** 2013-12-30

**Authors:** Richard A. Russell, David F. Garway-Heath, David P. Crabb

**Affiliations:** 1 Department of Optometry and Visual Science, City University London, United Kingdom; 2 NIHR Biomedical Research Centre for Ophthalmology, Moorfields Eye Hospital NHS Foundation Trust and UCL Institute of Ophthalmology, London, United Kingdom; Massachusetts Eye & Ear Infirmary, Harvard Medical School, United States of America

## Abstract

**Objective:**

To develop a model to simulate visual fields (VFs) in glaucoma patients, and to characterize variability of the Mean Deviation (MD) VF summary measurement using real VFs and simulations.

**Methods:**

Pointwise VF variability was previously approximated using longitudinal VF data (24–2 SITA Standard, Humphrey Field Analyzer) from 2,736 patients; these data were used to build a non-parametric model to simulate VFs. One million VF simulations were generated from 1,000 VFs (1,000 simulations per ‘ground-truth’ VF), and the variability of simulated MDs was characterized as a function of ground-truth MD and Pattern Standard Deviation (PSD).

**Results:**

The median (interquartile range, IQR) patient age and MD was 66 (56 to 75) years and −3.5 (−8.3 to −1.1) decibels, respectively. The inferred variability as a function of ground-truth MD and PSD indicated that variability, on average, increased rapidly as glaucoma worsened. However, the pattern of VF damage significantly affects the level of MD variability, with more than three-fold differences between patients with approximately the same levels of MD but different patterns of loss.

**Conclusions:**

A novel approach for simulating VFs is introduced. A better understanding of VF variability will help clinicians to differentiate real VF progression from measurement variability. This study highlights that, overall, MD variability increases as the level of damage increases, but variability is highly dependent on the pattern of VF damage. Future research, using VF simulations, could be employed to provide benchmarks for measuring the performance of VF progression detection algorithms and developing new strategies for measuring VF progression.

## Introduction

Glaucoma is the leading cause of irreversible blindness worldwide [1], affecting more than 70 million people [2]. The disease is characterized by damage to the optic nerve head and retinal nerve fibers, which can often be observed using a slit-lamp examination. Glaucoma is frequently, but not invariably, associated with raised intra-ocular pressure. Early detection is important for blindness prevention, and regular monitoring for deterioration (‘progression’) in vision is a fundamental aspect of clinical management. The extent of damage to the visual field (VF), which is the area of our vision in which objects can be seen, relates to the reduction of vision-related quality of life in glaucoma patients [3]–[5]. Glaucoma management aims to preserve the patient's vision and VF. Tests of vision, such as the VF test, are, therefore, of considerable clinical importance. VF testing (also known as perimetry) aims to locate damaged areas in a patient's field of vision using an automated machine that systematically measures the patient's ability to identify the presence of a small spot of light at different locations in their VF (‘contrast sensitivity’). Interpretation of results from standard automated perimetry (SAP) is challenging because VF measurements are very variable, as revealed by psychophysical experiments using frequency-of-seeing (FOS) procedures [6]–[8] and test-retest clinical studies [9]–[16]. Variability of SAP measurements necessitates frequent monitoring and/or a long period of time to accurately detect true disease progression [17] [18].

There is no perfect technique for diagnosing glaucoma or monitoring the disease. The lack of a definitive measurement makes it very difficult to gauge the performance of instruments, such as SAP, to evaluate glaucomatous progression. In particular, *quantitative* assessment of any algorithm or test requires access to ground truth, which is not available for the measurement of contrast sensitivity in SAP. Hence, computer simulation provides a means for generating artificial VF data consistent with real results obtained by SAP. For over twenty years, simple computer simulations have been used to assess VF test strategies and contrast simulated results with real VF data [18]–[26]. Simulation provides a reproducible and adjustable way of investigating the behavior of cross-sectional and longitudinal VF data in large volumes. In a recent study, we explored the relationship between VF variability and contrast sensitivity in VFs using a statistical method to quantify heteroscedasticity in longitudinal data [27]. We investigated almost 15,000 VFs from over 2,700 patients tested in standard clinic conditions. Approximately one million residuals were extracted to characterize VF variability by sensitivity level by fitting a linear model of pointwise sensitivity measurements (measurements of contrast sensitivity at different locations in the VF) against time of follow-up. The residuals associated with each fitted-sensitivity level were used in the current study in order to build a novel non-parametric computational model for generating VF simulations.

Unlike previous VF simulations [18],[25],[26],[28], our computer model is non-parametric and simulates VF sensitivity using empirical estimates of VF variability. In addition, previous simulations [18],[25],[26] have been based on estimates of variability in just tens of subjects in FOS studies [7], while our model is based on VF variability in almost 3,000 clinic patients, constituting almost one million data points. In this study, we describe the model and apply it to generate one million VF simulations from 1,000 ‘ground-truth’ VFs (1,000 simulations per real VF). These simulations were then analyzed to examine the variability associated with the ‘Mean Deviation’ (MD) VF summary measurement, as a function of the level of MD [29]. The MD is a summary statistic, which measures the mean increase or decrease of a patient's overall VF compared to a person with healthy vision of the same age. Another summary statistic, ‘Pattern Standard Deviation’ (PSD), measures the irregularity of patients' VFs. Large PSD values tend to indicate localized damage; however, patients with severe damage throughout their VF will have a relatively small PSD measurement. Mean Deviation and PSD are routinely used in clinical practice to identify glaucomatous defects and track progression of the disease. Nevertheless, the relationship between the levels of MD and PSD and the variability of MD is unclear. Some reports suggest that MD variability is non-stationary, increasing with disease severity in glaucoma patients [30]–[32]. However, no research to date has reported on the influence of pointwise VF variability, and therefore, the pattern of VF damage, on the variability of MD. A better understanding of MD variability will help clinicians to decipher glaucomatous VF test results and assess progression of this index.

## Materials and Methods

### Ethics statement

Patients' data was anonymised prior to investigation and did not contain personal or sensitive information. As such patients' written consent for their data to be used in the study was not required. The study adhered to the tenets of the Declaration of Helsinki and was approved by the research governance committee of City University London, United Kingdom.

### Study sample: VF variability

**Figure 1:**
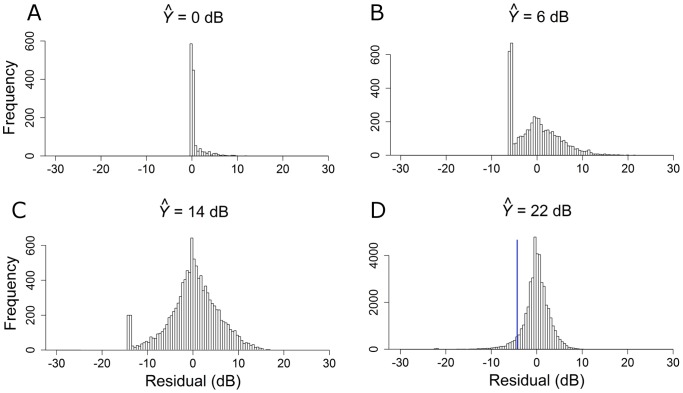
Distributions of VF variability according to sensitivity levels (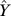): 0, 6, 14 and 22 dB. The blue line in the bottom right plot illustrates a single simulated draw from this distribution.

The study sample used to derive pointwise VF variability, which forms the foundation of the simulation model, is described in detail elsewhere [27]. In summary, a retrospective analysis of 14,887 anonymised VFs from 2,736 eyes from 2,736 patients (one eye randomly selected from each patient) attending the Glaucoma Clinics of Moorfields Eye Hospital, London between 1997 and 2009 was conducted. All VFs were carried out with the Humphrey Field Analyzer (HFA; Carl Zeiss Meditec, Dublin, CA) using the 24–2 test pattern with a Goldmann size III target and the SITA Standard testing algorithm. The median (interquartile range) follow-up was 6 (5 to 7) VF tests spanning 5.5 (3.9 to 7.0) years. The relationship between variability and measured-sensitivity was analyzed by examining the residuals from linear regression of pointwise sensitivity over time. Residuals from pointwise linear regression of sensitivity (the 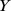 variable in decibels (dB)) against time (the 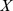 variable in years) were examined for each eye's series of VFs (2,736 eyes) using ordinary least squares linear regression (OLSLR) and Tobit linear regression (TLR).[33] The residuals extracted from linear regression were binned into single dBs bins in the range [0 to 36] dB according to the fitted-sensitivity value rounded to the nearest whole decibel; see Figure 1. The residuals from OLSLR were then used to simulate pointwise VF sensitivity in our model, described next. All test locations of a given patient were included in the simulations.

### Visual field simulations

**Figure 2:**
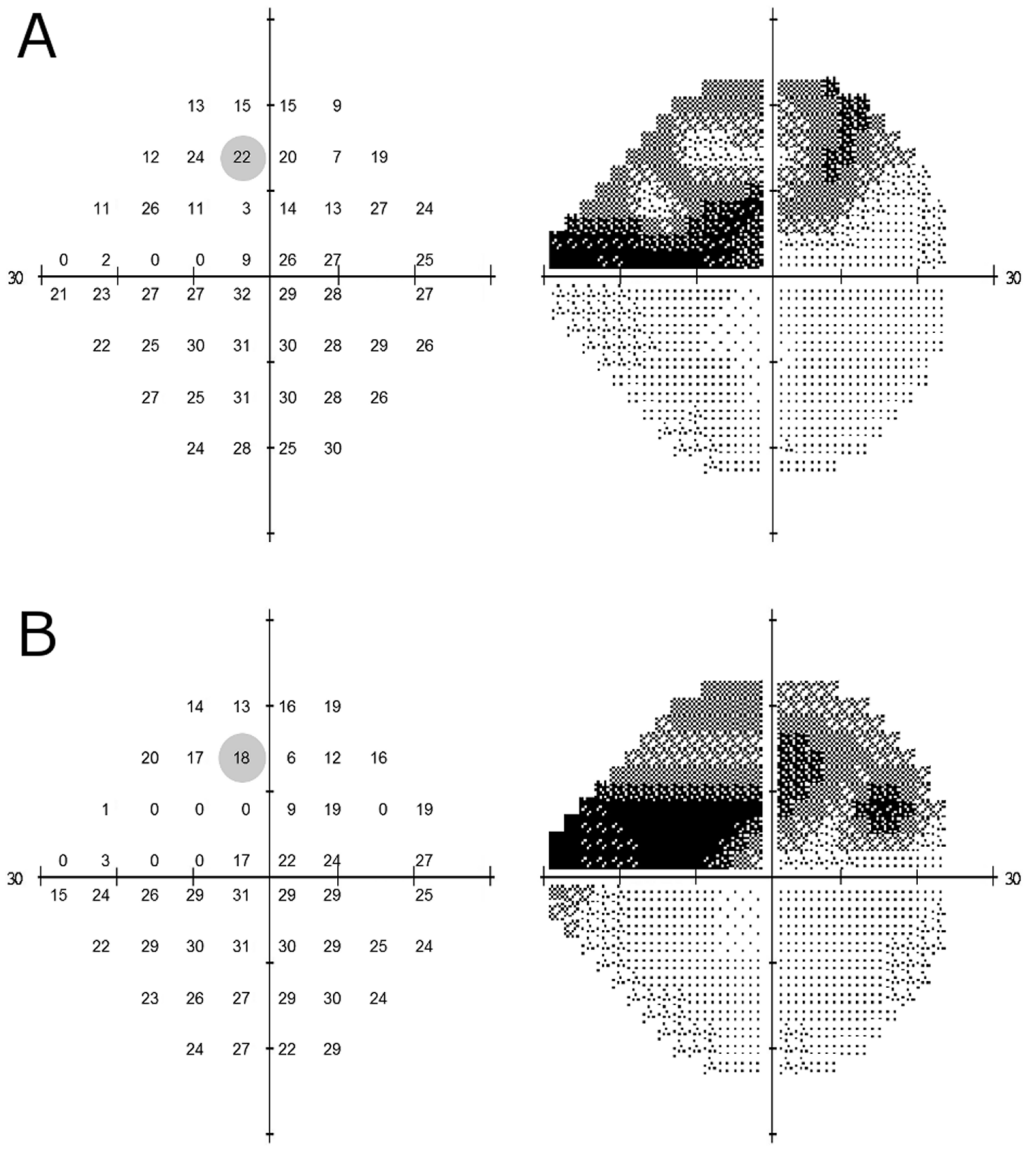
Ground-truth VF (A). The left hand grid of numbers illustrates pointwise sensitivity for all 52 locations in the VF, while the right hand image shows the corresponding grayscale plot. The variability of the shaded VF point (sensitivity equal to 22 dB) is indicated in the bottom right distribution in Figure 1. **Simulated VF (B) derived from ground-truth VF shown in panel A.** The left hand plot illustrates simulated pointwise sensitivities for all locations in the above VF, while the right hand image shows the corresponding grayscale plot. The shaded VF point (sensitivity equal to 18 dB) was derived by the simulated draw shown in the bottom right distribution in Figure 1.

The residuals extracted from OLSLR of longitudinal pointwise VF sensitivity in [27] underpin our VF simulation model; given a ‘true’ sensitivity value, they tell us the range of measured-values expected for any given test – thus allowing VF pointwise sensitivity to be simulated. For example, to simulate VF sensitivity when the ‘true’ value is equal to 22 dB, we randomly sample from the distribution of residuals associated with a fitted-sensitivity of 22 dB. The simulation is demonstrated in the bottom right distribution of Figure 1 (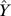 = 22 dB); in this plot approximately 50,000 residuals are associated with an OLSLR fitted-sensitivity bin of 22 dB while the blue line signifies the result from randomly drawing a single residual from the distribution. In this simulation, the VF sensitivity at that one location would be equal to 18 dB because the sampled residual was equal to −4.07 dB. In order to simulate an entire VF test, sensitivities are simulated one-by-one for each point in the entire VF; see Figure 2. Next, MDs and PSDs were calculated by first transforming raw sensitivities to total deviation values using published normative values describing the relationship between age and sensitivity in healthy individuals [29]. The MD was then calculated as the weighted mean of the total deviation values, with weights equal to the inverse of the variance observed at each VF location in the healthy reference group; as shown in Figure 3 of [29]. The PSD, on the other hand, was calculated as the weighted standard deviation of the total deviation values, with weights also derived from Figure 3 of [29]; this is identical to taking the weighted standard deviation of the pattern deviation values. Blind VF points (displayed as “<0” in the HFA printout) were coded as 0 dB.

**Figure 3:**
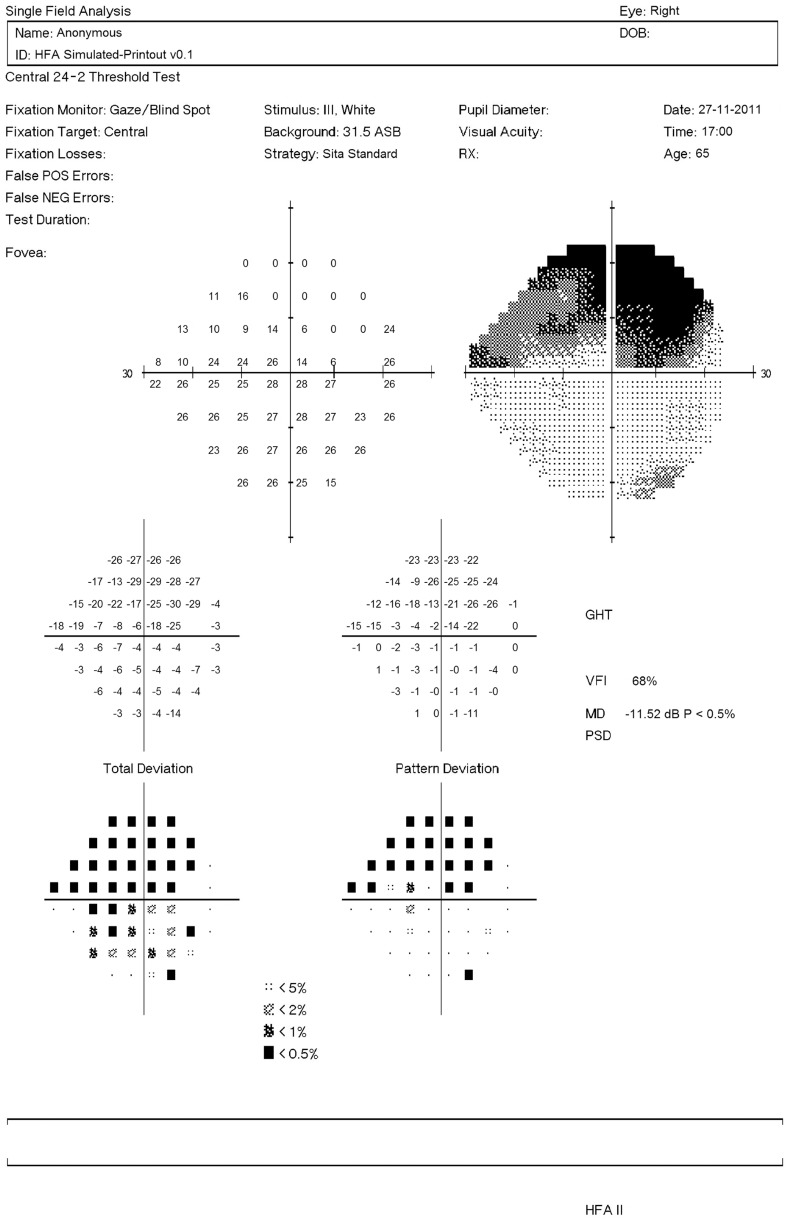
Simulated VF shown as a HFA-like printout. The grid of numbers in the top left represent the simulated sensitivities; while the adjacent grayscale plot provides a graphical representation of the VF (darker areas represent defects). The number grids below represent the difference in the patient's VF sensitivities and those of a healthy individual of the same age: without correction for a general reduction in retinal sensitivity (‘Total Deviation’); and with correction (‘Pattern Deviation’). Below these two grids are probability maps, which indicate whether the reductions in sensitivities are significant.

One thousand simulated VFs were generated from each of 1,000 patients' VFs (1,000 eyes) visiting Moorfields Eye Hospital between 1997 and 2009. These patients were not included in the analysis to establish pointwise VF variability in [27]. All VFs were carried out with the HFA using the 24–2 test pattern with a Goldmann size III target and the SITA Standard testing algorithm. Simulated pointwise VF sensitivities were generated via random sampling from the corresponding distributions of residuals for each ‘true’ sensitivity; the process was iterated for all 52 VF test points (excluding locations in the blind spot) in order to generate 1000 simulated VFs for every ground-truth VF. Finally, entire HFA-like VF printouts including greyscales were generated using custom-written R code; see Figure 3.

All statistical analyses and computational work (including VF simulations) were carried out using custom-written software in the open source programming language, R [34].

## Results

Table 1 summarizes the characteristics of the 1,000 patients that make up the 1,000 ground-truth VFs.

**Table 1:** Characteristics of glaucoma patients' ground-truth VFs.

Measurement	Median (interquartile range)
Age	66.0 (55.8 to 75.5) years
Mean Deviation	−3.5 (−8.3 to −1.1) dB;2.5th to 97.5th percentile: −22.0 to 1.4 dB
Pattern Standard Deviation	2.8 (1.7 to 6.8) dB;2.5th to 97.5th percentile: 1.2 to 12.1 dB
Pointwise sensitivity	27 (22 to 30) dB

Measurement variability in pointwise VF sensitivity varies significantly with the level of measurement; variability is small at high sensitivity levels, but markedly increases as sensitivity decreases to a level of 10 dB, where residuals span almost the entire measurement range of the instrument. At sensitivities less than 10 dB, the observed reduction in variability can be explained by the limited measurement range of SAP (0 to 50 dB), as revealed by the negative skew in the distributions at 0 dB and 6 dB levels in Figure 1.

**Figure 4:**
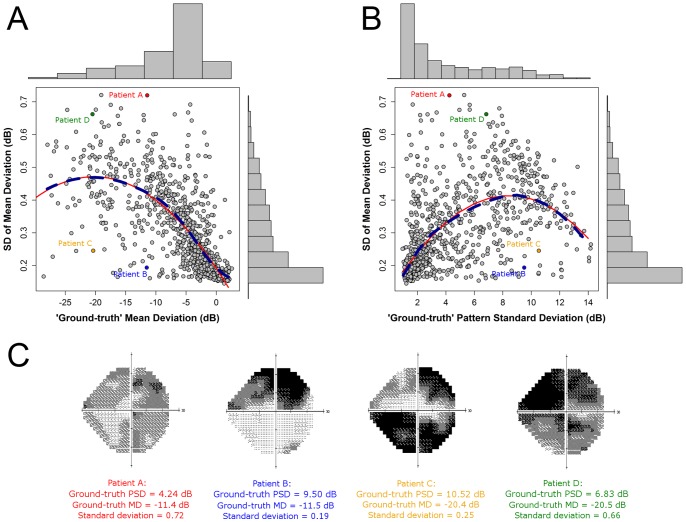
Mean Deviation variability according to level of damage, and grayscale plots for four ground-truth VFs. The MDs of Patients A–D are −11.4, −11.5, −20.4 and −20.5 dB, respectively, while the corresponding standard deviations of MD are 0.72, 0.19, 0.25 and 0.66, respectively.

We investigated the relationship between MD variability, the levels of MD and PSD, and the pattern of pointwise VF damage, through simulation. Figure 4 shows the variability of MD, according to the standard deviation (SD) of 1000 simulated VFs (MDs), as a function of the ground-truth level of MD (see Figure 4A) and PSD (see Figure 4B). The dashed dark blue lines in Figure 4 indicates the locally weighted polynomial regression [35] (‘LOESS’ regression), which gives an indication of how variability changes, on average, with the change in level of MD. The red lines, on the other hand, illustrate the results of fitting a second order model with a quadratic predictor: MD in Figure 4A (adjusted R^2^ = 0.55) and PSD in Figure 4B (adjusted R^2^ = 0.38). Figure 4A suggests that variability tends to increase as the level of MD reduces, with some evidence that variability peaks around −20 dB. Figure 4B suggests that variability tends to increase as the level of PSD increases, with some evidence that variability peaks around 8 dB. For VF loss associated with early glaucoma, where a significant amount of MD loss is approximately defined as −2 dB, variability is half that observed when MD is equal to −10 dB, which corresponds to VF loss associated with moderate glaucoma [36]. Interestingly, as demonstrated by the considerable scatter in Figure 4A and 4B, the standard deviation varied more than three-fold between patients with approximately the same levels of ground-truth MDs, from about 0.2 dB to over 0.7 dB (see Patients A and B in Figure 4C).

## Discussion

Standard automated perimetry continues to be the yardstick for detecting and monitoring glaucoma in clinical practice and clinical trials of new therapies for the disease. Nonetheless, SAP is affected by factors including learning effects and patient fatigue [37],[38]. Measurement variability is also induced by estimation errors associated with testing strategies, such as staircases, used in clinical SAP [21],[24]. Several studies have shown that a reduction in VF sensitivity is accompanied by an increase in response variability [9]–[16]. This combination of issues leads to considerable difficulties in differentiating true VF change from inherent noise, making glaucoma management and treatment decisions very challenging.

Characterizing properties of VF measurements and assessing the performance of VF progression algorithms requires an independent gold-standard, which is not available for retinal contrast sensitivity. This problem is widely recognized in glaucoma research and many substitute approaches of classifying ‘true’ glaucomatous progression have been attempted [24],[39]. Visual field models offer an alternative benchmark and permit large amounts of data to be simulated, with known characteristics, for subsequent analysis. In this way, the importance of different variables on VF measurements can be identified. Visual field simulations have previously been used in glaucoma research to evaluate testing strategies and progression detection tools [18]–[25],[40]. However, earlier models have been based on limited VF data, and so may not accurately reflect clinical VF measurements. For instance, some models [18],[25],[26],[28] have been based on a linear equation for VF variability in [7], which was based on FOS curves with absolutely no data below 10 dB. Furthermore, these previous models have assumed that variability is Gaussian-distributed. Conversely, our simulations are based on SAP results from thousands of clinic patients, and empirical estimates of VF variability. Importantly, our simulations do not model VF variability as a Gaussian distribution, which is clearly not appropriate from inspection of Figure 1. In particular, Figure 1A demonstrates that VF sensitivity is truncated at 0 dB (hence a lack of negative residuals), which is also evident in Figures 1B and 1C, and to some extent in Figure 1D. Nevertheless, it is important to note that our VF simulations do not include variables that can account for factors such as patient fatigue, learning effects, test reliability, technician experience, time of day, and seasonal effects [37],[38],[41]. Thus, the magnitude of variability simulated may be less than observed in clinical practice due to these other factors. It is also worth noting that our model is based on empirical estimates of VF variability derived from linear modeling of VF decay, which is only an approximation to ‘true’ VF variability.

Mean Deviation is routinely used to summarize overall VF damage in individual patients, as well as in groups of patients enrolled in clinical studies [30]. Since the MD index is a weighted average, it is less sensitive to localized glaucomatous damage in the VF; however, as a summary statistic, the MD is robust to measurement noise at individual locations. Previous research suggests that MD variability increases as the level of MD decreases [30]–[32], but, until now, no study has investigated the impact of the pattern of VF damage on the variability of MD. In the absence of computer simulations, such a study would be very difficult to carry out using clinical data as it would be almost impossible to disentangle the relationship between patient error, algorithm error and other measurement errors.

We carried out computer simulations to investigate the association between MD variability, the levels of MD and PSD, and the pattern of VF damage. Figure 4A illustrates how MD variability increases, on average, as the level of MD decreases, with some evidence that variability peaks around −20 dB. Figure 4B demonstrates a strong association between MD variability and PSD values; larger PSD values, suggesting greater glaucomatous VF damage, are associated, on average, with higher variability. For VF loss associated with early disease (MD ≈−2 dB), variability is half that in moderate glaucoma (MD ≈−10 dB) [36]. More interestingly, variability varies more than three-fold between some patients with approximately the same levels of MDs, suggesting that the pattern of pointwise VF damage has a significant impact on MD variability. This is supported by examining the VFs of patients with roughly equivalent MD damage but very different levels of variability; see Patients A and B, and Patients C and D in Figure 4C. These grayscale VF plots indicate that VFs with global diffuse damage (Patients A and D) tend to be more variable than VFs with localised damage and other regions that are healthy (Patients B and C) despite the MD levels being almost the same. It is also interesting to note that the PSD values of these two pairs of patients hint at the differences in MD variability. For example, the PSD of Patient A's VF is less than half that of Patient B's yet the patients share roughly the same level of MD; the PSD of Patient A's VF is much smaller than that of Patient B due to the diffuse rather than localized damage seen in the VF of Patients A and B respectively. This highlights the importance of measuring VF progression using individualized rather than population-based criteria.

Recently, there has been renewed interest in the MD index. Junoy-Montolio et al. [41] showed that VF locations which test blind on consecutive tests may result in an underestimation of the MD index, which could affect MD-based progression detection algorithms [41]. Their results also suggest that censoring of pointwise sensitivity measurements at blind locations leads to a reduced dynamic range of MD, and therefore, reduced variability of this index. Our results are thus in good agreement with those from Junoy-Montolio et al., and we support their conclusion that VF points that have been shown to be reproducibly blind do not contribute to progression detection. Figure 4A suggests that, on average, this occurs at approximately −20 dB, since MD variability starts to decrease at this value. This is not surprising given that many points in the VF will likely be perimetrically-blind at this level of damage, leading to the shrunken dynamic range of MD described above. In addition, the −20 dB cut-off correlates with the point at which pattern deviation calculations are deemed unreliable and are not shown in HFA printouts [30]. Furthermore, Junoy-Montolio et al. showed that the number of VF points discarded by the HFA ‘Guided Progression Analysis’ software [42],[43] increases with disease progression up to an MD of about −20 dB but decreases beyond that point [41].

Wall et al. recently investigated the relationship of MD and its variability for Goldmann size III and size V stimuli [31]. They determined that the repeatability of size V MD was slightly better than size III, but variability increased with increasing damage for both stimuli. Depending on the size of the stimulus and the amount of VF damage, a change of 1.5 to 4 dB in MD is necessary to be outside normal 95% confidence limits [31]. Our results suggest that any confidence limits also need to consider the pattern of VF damage and not just the amount of VF damage. Wall et al. concluded their research by stating that “further work needs to be done to determine criteria for identifying visual field change”. We believe that VF simulations provide an excellent means by which to provide benchmarks for measuring the performance of VF progression detection algorithms and developing new strategies for measuring VF progression. For example, if assessing the rate of VF damage using linear regression of MD over time, it may be important to account for the non-stationary variability observed with disease worsening in the regression.

Visual field variability leads to false-positive diagnoses of progression when patients actually have stable glaucoma, which may lead to needless treatment changes and costs to both patient and healthcare provider [32]. Conversely, glaucomatous progression may be missed if clinicians deem any change is due to inherent measurement noise. In this study, computer simulations have allowed us to gain a better understanding of MD variability; on average, MD variability increases as the VF becomes more damaged. Thus, detecting progression in MD will tend to be more difficult in patients with moderate or advanced glaucoma than in patients with early disease. Consequently, it may be advantageous to carry out more frequent VF testing in glaucoma patients with worse MDs and/or diffuse damage. Progression detection is exasperated by the fact that MD slope variability is related to shorter follow-up duration – research suggests that the phenomenon of ‘positive slopes’ and ‘rapid progressors’ is partly due to too short series of VFs [44]–[46]. This study also suggests that clinicians should be aware that variability can vary more than three-fold between patients with roughly equivalent MDs; in particular, we observe that VFs with global diffuse damage tend to be more variable than VFs with localized damage and other regions that are healthy. This information may help clinicians to differentiate glaucomatous VF test results and assess progression of this index.
